# The role of metabolism (and the microbiome) in defining the clinical efficacy of dietary flavonoids^1^

**DOI:** 10.3945/ajcn.116.136051

**Published:** 2016-11-23

**Authors:** Aedín Cassidy, Anne-Marie Minihane

**Affiliations:** Department of Nutrition and Preventive Medicine, Norwich Medical School, University of East Anglia, Norwich, United Kingdom

**Keywords:** absorption, ADME, flavonoids, genotype, health, metabolism, microbiome

## Abstract

At a population level, there is growing evidence of the beneficial effects of dietary flavonoids on health. However, there is extensive heterogeneity in the response to increased intake, which is likely mediated via wide interindividual variability in flavonoid absorption and metabolism. Flavonoids are extensively metabolized by phase I and phase II metabolism (which occur predominantly in the gastrointestinal tract and liver) and colonic microbial metabolism. A number of factors, including age, sex, and genotype, may affect these metabolic processes. In addition, food composition and flavonoid source are likely to affect bioavailability, and emerging data suggest a critical role for the microbiome. This review will focus on the current knowledge for the main subclasses of flavonoids, including anthocyanins, flavonols, flavan-3-ols, and flavanones, for which there is growing evidence from prospective studies of beneficial effects on health. The identification of key factors that govern metabolism and an understanding of how the differential capacity to metabolize these bioactive compounds affect health outcomes will help establish how to optimize intakes of flavonoids for health benefits and in specific subgroups. We identify research areas that need to be addressed to further understand important determinants of flavonoid bioavailability and metabolism and to advance the knowledge base that is required to move toward the development of dietary guidelines and recommendations for flavonoids and flavonoid-rich foods.

## INTRODUCTION

Dietary flavonoids represent a diverse range of polyphenolic compounds that are present in many commonly consumed fruits, vegetables, grains, herbs, and beverages (1). Growing evidence from both population-based studies and randomized controlled trials (RCTs)^2^ suggests that several flavonoid subclasses may be important for cardiometabolic health with substantial interest in other outcomes, including cognitive function, Parkinson disease, and specific cancers, also developing (2–6). In this article, rather than conducting an exhaustive review of the current literature, we set out to summarize the current state of the art in the field by drawing on examples from recent studies on specific subclasses to highlight gaps in our understanding that may explain discrepancies in findings across the translational research pathway. We also identify research areas that need to be addressed to further understand how to optimize intake of flavonoids for different health benefits and in specific subgroups and to advance the knowledge base that is required to move toward the development of dietary guidelines and recommendations for flavonoids and flavonoid-rich foods.

**FIGURE 1:**
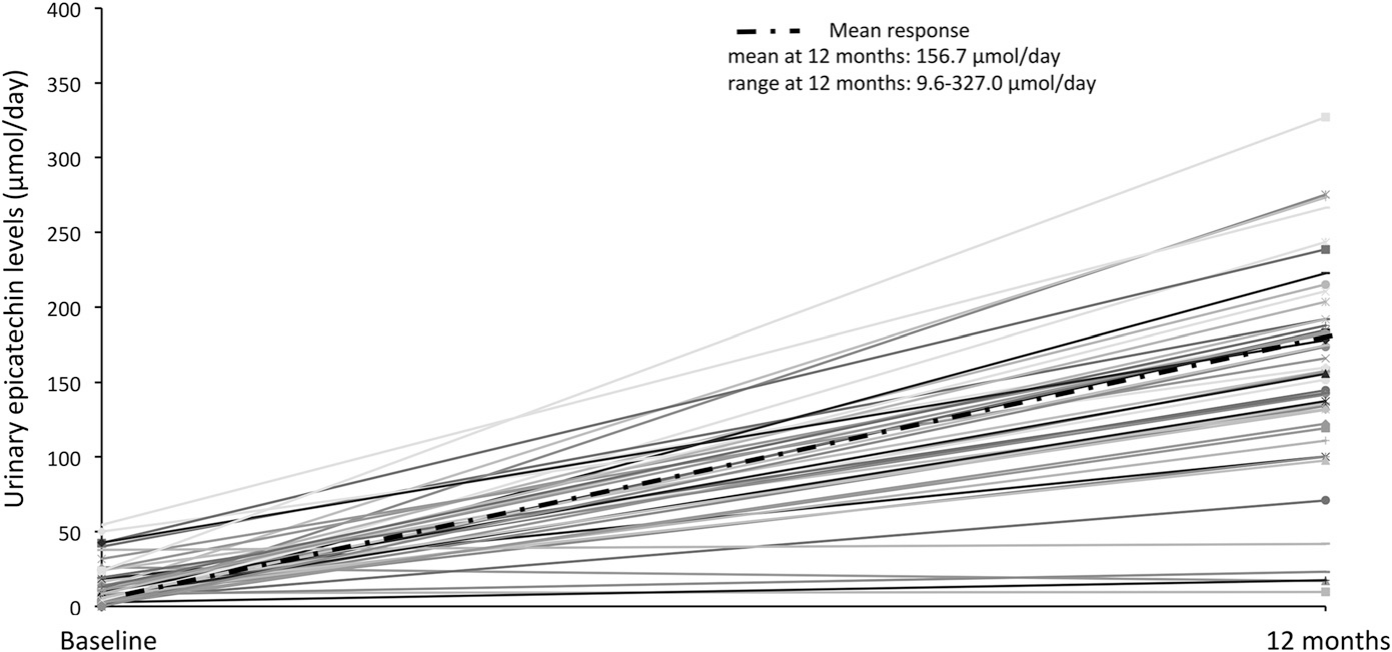
Mean interindividual variability in urinary epicatechin excretion in 93 participants after intake of 85 mg epicatechin/d for 1 y (12).

The structural complexity of flavonoids has led to their subclassification as flavonols, flavones, flavanones, flavan-3-ols (including their oligomeric and polymeric forms, proanthocyanidins), isoflavones, and anthocyanins (7–9). The diversity of flavonoid structures undoubtedly contributes to differences in biological efficacy with subtle differences affecting both bioavailability and bioactivity. It is clear that the bioavailability of dietary flavonoids is highly variable between individuals. After ingestion, flavonoids undergo extensive metabolization with absorption occurring in both the small and large intestines with a substantial fraction of intake reaching the colon, where the flavonoids are exposed to colonic microbiota. The resident microbiome operates as a metabolic reactor, thereby playing a key role in catabolizing unabsorbed flavonoids into smaller molecules such as phenolic and aromatic acids, which may become bioavailable (10). Data from available interventions provide evidence to suggest that there is extensive variability in the amount of metabolites that have been measured in biological samples, with 15–99% of the original flavonoid dose recovered as a wide range of flavonoid metabolites (7, 11). The heterogeneity has been highlighted in data from a 1-y flavonoid intervention in 93 participants; mean 24-h urinary epicatechin (flavan-3-ol) excretion rates were 156.7 μmol/d with wide interindividual variability that ranged from 9.6 to 327.0 μmol/d across study participants (**Figure 1**) (12). This metabolic variability has likely been a factor that has contributed to the wide CIs in the physiologic responsiveness observed in both intervention and observational studies.

**FIGURE 2:**
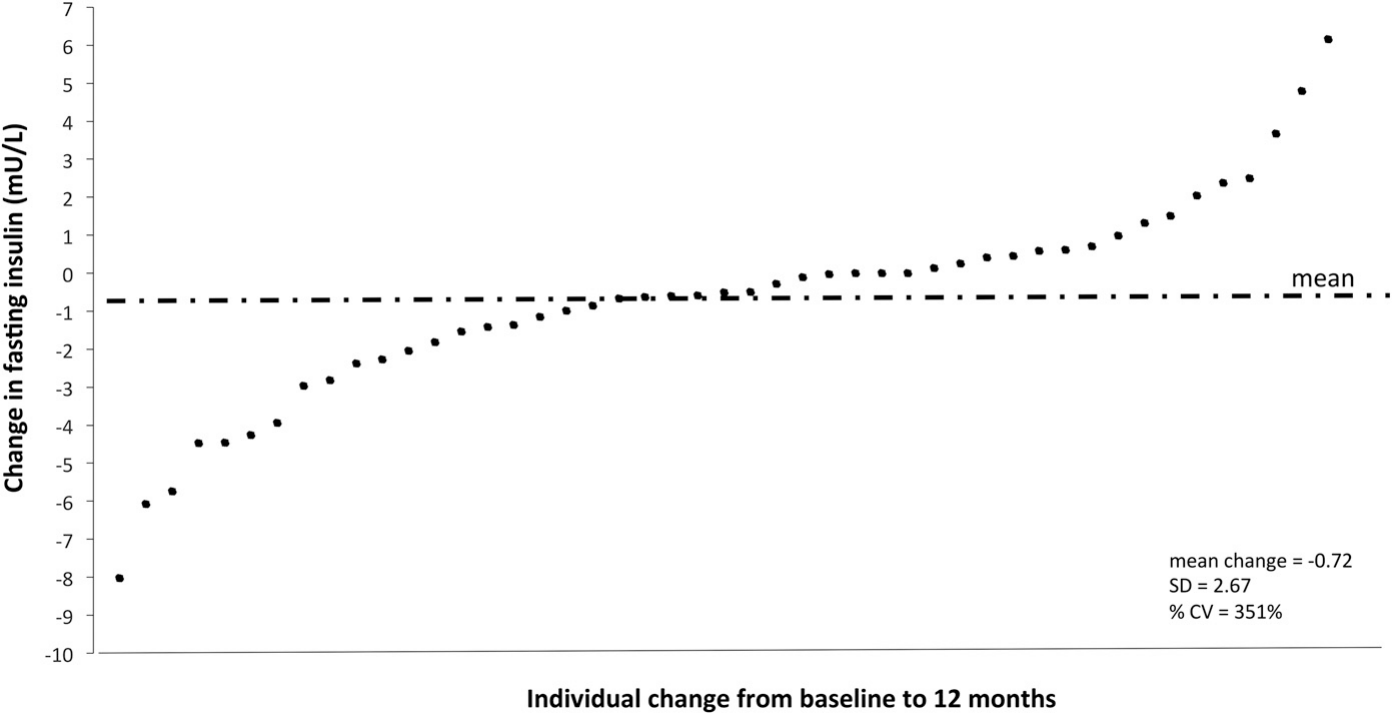
Variability in changes in fasting insulin concentrations (mU/L) in 93 participants after a 1-y flavonoid intervention (12).

Although a large variability in the physiologic response to flavonoid intake has also been observed (**Figure 2** shows the insulin response to the previously mentioned 1-y intervention) (12), RCTs have seldom concurrently addressed metabolism and health outcomes. The few studies that have included plasma or urinary measures of the flavonoid under study did so predominantly as a measure of compliance to the intervention (7, 9, 12). At a population level, the heterogeneity in responsiveness and a poor response to flavonoid intake in certain individuals may, therefore, obscure beneficial associations between intakes and health outcomes in responsive subgroups.

In some prospective studies, strong associations between low intakes of anthocyanins (median intakes of 15 mg/d with a range up to 1 g/d) and beneficial health effects have been observed (2, 3), whereas in other studies, no effects have been observed for anthocyanins, but benefits have been reported for other subclasses including flavonols (13, 14). This variability in the strength of the association between flavonoid intake, the wide CIs observed, and the responsiveness both within and between populations were likely, in large part, attributable to differences in the absorption, distribution, metabolism, and elimination (ADME) of flavonoids as will be discussed. This review will focus mainly on the current knowledge and research gaps for the main subclasses of flavonoids, including anthocyanins, flavonols, flavan-3-ols, and flavanones, for which there is growing evidence from prospective studies for beneficial effects on health. The isoflavone-related literature will not be included because isoflavone metabolism and biofficacy have been extensively reviewed previously (15, 16), and intakes of this flavonoid subgroup are low (<3 mg/d) in individuals who have followed a Western-style diet in which soy products are not commonly consumed (17).

## OVERVIEW OF FLAVONOID METABOLISM

**FIGURE 3:**
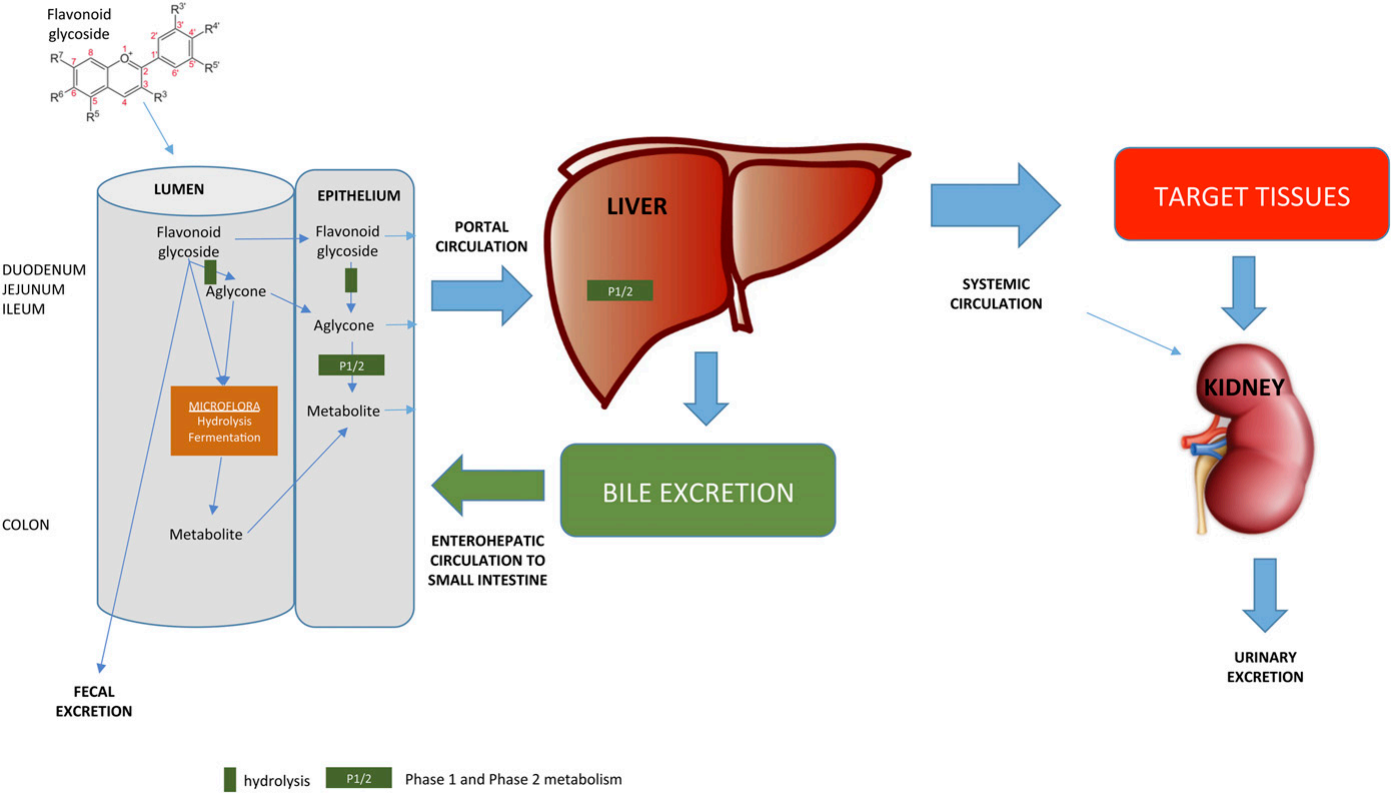
Overview of flavonoid absorption and postabsorptive metabolism.

An overview of flavonoid ADME is given in **Figure 3** (18–20). Flavonoids are generally consumed as glycosides with a proportion of the aglycone released either in the epithelium or lumen of the small intestine. Unlike dietary macronutrients and micronutrients, a large proportion of ingested flavonoids are unabsorbed in the proximal intestine and reach the colon where they are exposed to microbiome-mediated hydrolysis and fermentation. Within the epithelium, flavonoids undergo phase I metabolism with the resultant metabolites transported to the liver via the portal vein. In the liver, they undergo further phase I and phase II metabolism that result in more-polar compounds, which mediate an array of biological effects in target tissues. The efflux of flavonoids from the body is via the kidney, from the intestinal epithelium, and via bile excretion. Flavonoids secreted via the biliary route into the duodenum are subjected to the action of microbial enzymes and may be reabsorbed and undergo enterohepatic recycling (Figure 3).

### Absorption

In the lumen of the small intestine, lactase phlorizin hydrolase (LPH, lactase) hydrolyses flavonoid glycosides into their respective aglycones (21). LPH is a transmembrane protein with broad substrate specificity for a range of flavonoid-*O*-β-d-glucosides. Aglycones may enter the epithelial cells by passive diffusion as a result of increased lipophilicity. Alternatively, the glycosides can be directly transported into the epithelium via epithelial transporters such as sodium-dependent glucose transporter (21–24) with the glycosides subsequently hydrolyzed by intracellular β-glucosidases such as cytosolic β-glucosidases (21, 25, 26). Therefore, as a general rule, flavonoid glycosides are cleaved either in the intestinal lumen or epithelium before absorption. However, anthocyanins are an exception and are present in plasma and urine as glycosides (27). The impact of lactase deficiency (which has a 60–100% prevalence in many Latin American, African, and Asian countries) on flavonoid bioavailability is currently unknown.

Membrane-bound ATP-binding cassette (ABC) transporter proteins are involved in the epithelial transcellular passage of many compounds including dietary flavonoids (28). This protein group is involved in the efflux of bioactive compounds either through the basolateral membrane into the portal bloodstream, which facilitates absorption, or transported back into the intestinal lumen thereby reducing bioavailability. The main ABC-group members include P-glycoprotein, multidrug resistance proteins, and breast cancer–resistance protein (28). In addition to transcellular absorption, transport via the paracellular route has also been identified (29, 30) with its relative contribution to the overall bioavailability likely to be dose and isoform dependent.

### Postabsorptive metabolism

After absorption, flavonoids may undergo phase I metabolism in the liver (oxidation or *O-*demethylated) by cytochrome P450 monooxygenases. In humans, there are 57 cytochrome genes that are divided in 18 families with isoforms such as cytochrome 1A1, cytochrome 1A2, cytochrome 1B1, cytochrome 3A4 (the predominant human intestinal and hepatic cytochrome 450s), and cytochrome 2C9 that are involved in flavonoid metabolism (31, 32). However, phase I metabolism–derived oxidation products tend to be minor metabolites of most flavonoids, which is probably due to the rapid glucuronidation, sulphation, or methylation of potential phase I substrates in the intestine and the liver (18, 33) by phase II conjugating enzymes including urine-5′-diphosphate glucuronosyltransferases (UGTs), sulphotransferases, and catechol-*O*-methyltransferases (COMTs). The glucuronide sulfate and methyl conjugates are more-polar metabolites and may be excreted via the kidneys in urine or via bile or transported by ABC-mediated efflux back into the intestinal lumen. In general, the majority of conjugates in the plasma and urine are glucuronides (34). The conjugation mechanisms are highly efficient, and aglycones are generally either absent in the circulation or present in low concentrations after physiologic intakes.

UGTs catalyze the transfer of a glucuronic acid from UDP-glucuronic acid to polyphenols (including flavonoids) and other xenobiotics. The *UGT* gene superfamily gives rise to >22 UGT isoforms that belong to the UGT1A, UGT2A, UGT2B, UGT3, or UGT8 families (35). The glucuronidation of flavonoids is regiospecific and isoform dependent (36, 37). Sulphotransferases add a sulfate moiety to the flavonoids. They also belong to a gene superfamily with >10 different sulphotransferase isoforms in humans (38, 39). Sulphotransferases 1A1–4 and 1E1 have been specifically shown to be involved in the metabolism of flavonoids (37, 40–44). COMTs are involved in the *O*-methylation of catecholic polyphenols including catechins, epicatechins, and epigallocatechins from the flavan-3-ol subclass (45). Methylation decreases the hydrophilicity of compounds, and after methylation, subsequent glucuronidation and sulphation are often needed for the effective elimination from the body.

### Tissue uptake

Although likely to be important determinants of flavonoid ADME and their effects on cell and tissue functions, little is known about the tissue uptake and subsequent partitioning of flavonoid metabolites. A limited number of studies that have focused mainly on anthocyanins have indicated that bilitranslocase may be involved in vascular epithelial and hepatic flavonoid uptakes and anthocyanin absorption from the stomach (46–49). However, the relative importance of bilitranslocase and the identity of other transporters remain to be established.

### Elimination

Breast cancer–resistance protein, organic anion transporting polypeptide, and, in particular, organic anion transporters 1 and 3 that are expressed in basolateral membranes of renal tubules and couple with phase I and phase II metabolism are thought to be important facets of the elimination process (28, 50–52). The overexpression of organic anion transporter is associated with more-efficient renal uptake and elimination into the urine (51). In addition to urine, a significant proportion of bioavailable flavonoid metabolites may be excreted via bile into the feces although enterohepatic recirculation results in some recycling back to the small intestine through bile excretion (53, 54).

## ETIOLOGY OF THE HETEROGENEITY IN FLAVONOID ADME

As detailed above, ∼200 proteins have been identified as having the potential to affect flavonoid ADME. Modulators of the expression and activity of these proteins, such as age, sex, and genotype, are likely, to varying degrees, to influence the circulating concentrations, elimination and tissue exposure to flavonoids (Figure 1) and, ultimately, to dose-response relations (Figure 2). Although little relevant published literature is currently available, lessons may be learned from traditional xenobiotic and, in particular, drug metabolism because many phase I and II metabolic pathways are common to both drug and flavonoid groups. While there is likely to be considerable redundancy in oxidation, glucuronidation, sulphation, and methylation pathways, there exists some evidence that flavonoid intake may influence drug metabolism with the reciprocal relation also likely to exist, with habitual drug use potentially influencing flavonoid ADME in an individual. Although currently largely unknown, this effect may be a particular issue in cases in whom the metabolism of the drug or flavonoid is reliant on one or a limited number of cytochrome, UGT, sulphotransferase, or COMT isoforms or in a situation of compromised phase I and II metabolic capacities, which are perhaps associated with disease or aging. Furthermore, it is also unclear what the physiologic consequences of altered ADME are likely to be. Reduced absorption would be predicted to reduce biopotency, but reduced phase I and phase II metabolism, although potentially reducing excretion rates and increasing the dose and length of tissue exposure, may result in a lower formation of more bioactive metabolites (relative to their parent compounds) and also potentially result in toxicity in susceptible individuals.

### Impacts of age and sex on xenobiotic and flavonoid ADME

The aging process is associated with reduced hepatic perfusion and morphology including reduced hepatocyte density, which has been suggested to reduce the phase I and phase II metabolism of xenobiotics (55, 56) and therefore potentially flavonoid metabolism. However, the impact of aging on the activity of oxidation and conjugation enzymes per se is controversial. In isolated perfused livers from 3- to 6- or 22- to 24-mo-old rats exposed to *p*-nitrophenol, there was evidence of reduced oxidation and glucuronidation with aging, which was speculated to be partly attributed to reduced cofactor availability (57). In contrast, no obvious effect of aging was reported in human (58) or rat (59) microsomal UGT activities in response to commonly prescribed medications. In more-recent publications, the individual or interactive effects of aging and sex on a wider range on xenobiotic metabolizing enzymes were examined. Fu et al. (60) included a gene-expression analysis of 101 xenobiotic-processing genes including cell transporters, phase I and II enzymes, efflux transporters, and transcription factors that were quantified at 10 time points in the livers of male and female mice across the life span (60). Complex impacts of both age and sex emerged, whereby the messenger RNA concentrations for 44% of the genes changed in male mice, and 63% of the genes changed in female mice, according to age. Significant upregulation and downregulation were evident, but overall, 40% of the xenobiotic-processing genes were lower in aged male mice and 43% in aged female mice. Kawase et al. (61) noted higher breast cancer–resistance protein, organic anion transporting polypeptide 1a1, and UGT1A1 and lower cytochrome 3A1 and cytochrome 32 in young female rats than in male rats, with an age-related downregulation of gene expression that was evident only in female rats (61). Two recent publications have specifically focused on flavonoids as model compounds. The glucuronidation characteristics of the flavonoid glucoside tilianin and its aglycone acacetin (flavones) were characterized with the use of human UGT isoforms, liver microsomes, and intestinal microsomes that were obtained from different animal species. Overall, and consistent with Kawase et al. (61), higher glucuronidation rates attributed to higher UGT1A1 activities were evident in females across several species (62). The impact of aging on the glucuronidation of quercetin and genistein in male rat hepatic microsomes was also studied (63). Overall, although some age-related changes were evident, they were modest in their magnitudes. The glucuronidation of genistein decreased with age. The quercetin total glucuronidation capacity was constant with age, but young and old rats had different metabolite profiles.

Therefore, although there is limited evidence to suggest that there is possibly higher UGT1A-mediated glucuronidation in females than in males and a possible overall age-related decline in phase I and phase II metabolism, available data are wholly inadequate to make any definitive conclusion regarding the likely impact of these variables on flavonoid metabolism in humans.

### Impact of genotype on xenobiotic and potentially flavonoid metabolism

As with the majority of phenotypes, it is likely that ≥50% of the interindividual heterogeneity in flavonoid ADME is attributable to genetic variability. The most recent output from the 1000 Genome Consortium, which was published in October 2015 (64), indicated that there are typically 88 million variants in a human genome, and with knowledge that the penetrance of individual variants is influenced by a range of behavioral, physiologic, and epistatic (gene × gene interactions) factors, the identification of which factors influence flavonoid ADME represents a major challenge. To date, the limited investigations have taken a candidate-gene approach and have focused on one or a small number of variants in a gene encoding for key phase I or II proteins.

Although currently completely unknown, because of the key role of LPH and β-glucosidases (see Absorption) in the initial hydrolysis of flavonoid glycosides, it is likely that variants in these loci may be important determinants of the bioavailability of the majority of flavonoid subclasses from the small intestine.

Perhaps the most extensively studied genotype that is relevant to flavonoid ADME is the *COMT* missense mutation (rs4680) with a *G-to-A* base change that results in a valine-to-methionine amino acid substitution at position 158 of the protein. This polymorphism is thought to produce a less stable protein, which in vitro studies have proposed can result in a 40% decrease in enzyme activity (65) and can influence the metabolism of a number of exogenous and endogenous compounds including catecholamines and a range of drugs (66). In a case-control study of Asian-American women, the consumption of green tea, which is rich in flavan-3-ols, was associated with reduced breast cancer risk with the strongest association evident in subjects with a low-activity *COMT A* allele (67). In a cross-sectional analysis of a subset of the Shanghai Cohort, the *AA* genotype had significantly lower urinary total polyphenols and concentrations of 3 of the 5 specific tea polyphenol metabolites [(−)-epigallocatechin, 4′-methylepigallocatechin and 5-(3′,4′,5′-trihydroxyphenyl)-γ-valerolactone] relative to the *GG* and *GA* groups with a trend for genotype-associated differences in epicatechin and 5-(3′,4′-dihydroxyphenyl)-γ-valerolactone (45). Consistent with these findings, in participants who were prospectively recruited according to genotype, urinary methylated epigallocatechin concentrations were significantly higher in the *GG* COMT group than in *AA* homozygotes after acute consumption of green-tea extract (68). In the Minnesota Green Tea Trial, overweight and obese postmenopausal women underwent a 12-mo intervention that examined the impact of green-tea extract on adiposity and measures of cardiometabolic health (69). A response to the intervention was established according to *COMT* genotype status with no overall impact of the intervention and no genotype × treatment interactions observed. However, no data on plasma or urinary catechin concentrations were reported, which would have allowed for the examination of the impact of the interindividual variability in metabolism on physiologic responses.

UGTs glucuronidate bilirubin, estrogens, and exogenous compounds, including dietary carcinogens and prescribed medications. The effects of *UGT* genotypes on the endogenous concentrations of these compounds, incidences of associated cancers, and responses to select drugs have been reported (70–81). An et al. (70) examined the impact of 6 single-nucleotide polymorphisms (SNPs), including 3 SNPs in the *UGT1A1* gene, on the daily warfarin dosage. One *UGT1A1* SNP (rs887829) exhibited significant association with warfarin use with *T*-allele carriers requiring higher doses than for individuals with the *CC* genotype (6.3 compared with 5.2 mg/d, respectively). In a study of 1600 colorectal cancer patients and 2500 unaffected siblings, the variation in 4 UGT genes (*UGT1A3, UGT1A6, UGT2B4*, and *UGT2B15*) modified risk of colorectal cancer either independently of interactively with nonsteroidal anti-inflammatory use (79). Variants in the promoter of the *UGT1A1* gene (UGT1A1*28, rs8175347), which result in 5, 7, or 8 repeats instead of 6 thymine-adenine repeats, were associated with decreased *UGT1A1* transcription and higher serum bilirubin with increased numbers of thymine-adenine repeats (75, 82). Several dietary phytochemicals, including flavonoids, have been shown to induce UGT1A1 activity (83, 84), with Lampe and coworkers reporting that the impact of the UGT1A1*28 genotype on bilirubin metabolism was modified by increased intakes of cruciferous vegetables, citrus fruit, and soy (72, 76, 78). Although intuitively, because of the role of UGT1A1 in flavonoid glucuronidation, the UGT1A1*28 variant is also likely to be an important modulator of flavonoid metabolism, its impact is currently unknown but worthy of investigation.

Because ≤10% of flavonoids are sulfated, variants in sulphotransferase genes may also affect flavonoid plasma and urinary profiles. Genetic variants in sulphotransferases with associated functional consequences have been identified with SNPs in sulphotransferases 1A1 and 2A1, which are associated with altered drugs responses and sex-steroid concentrations (18, 43). Cytochrome 3A4 is the most abundant isoform of cytochrome P450 in the adult human liver, with common *CYTOCHROME-3A4* variants that have been shown to influence testosterone metabolism (85). However, as with *UGTs*, the effects of *SULPHOTRANSFERASES* and *CYTOCHROME* and cytochrome genotypes on flavonoid metabolism remain to be tested.

Overall, there is a dearth of information on the genetic determinants of flavonoid metabolism. The previously discussed literature on variants of phase I and II genes that influence the metabolism of an array of endogenous and exogenous compounds may help inform future research in the flavonoid field. However, a justification for the selected gene-variant targets has been rarely provided with the functional consequences of genotype often unknown. Future studies should adopt a more genome-wide approach or targeted genotyping that is focused on the key enzymes specific to flavonoid (rather than drug) metabolism. The selection of which individual variants to assess is a challenge with an intuitive focus on exon variants and, in particular, nonsynonymous SNPs or those in gene-promoter regions that have the potential to exclude potentially highly functional variants in intron regions. Although relatively expensive, genome-wide association studies or whole-gene or -genome sequencings represent a more efficient approach to identifying genotypes that are important in flavonoid ADME and, therefore, potentially bioefficacy. Once a potentially functional genotype has been identified by untargeted approaches, its role should be subsequently confirmed with the use of a prospective recruitment according to the genotype approach in human volunteers along with the use of rodent or cell models in which the native gene has been replaced by human variants to establish the effects of the genotype on enzyme activity and flavonoid ADME.

### Potential impact of prescribed medication use on flavonoid metabolism

Because flavonoids and many prescribed medications share phase I and II metabolic processes, the effects of dietary flavonoid (and other bioactive) intake on drug ADME and dosing amounts have been of research and clinical interest for several decades (86–89). The impact of grapefruit consumption on cytochrome-3A4 activity and the metabolism of a large number of drug groups, such as calcium channel antagonists β-hydroxy-β-methylglutaryl–CoA reductase inhibitors and antihistamines, represents a widely cited example (90, 91). Although unknown, it is likely that drug use affects flavonoid metabolism and, ultimately, tissue total and metabolite flavonoid exposure and dose-response relations. The multiplicity of transferases with overlapping substrate specificity and likely considerable redundancy in phase I and II metabolic capacities may mean that there is little impact of single drug use on flavonoid metabolism in the majority of the population. However, in select subgroups, such as older adults who commonly consume a drug cocktail and may experience age-related declines in metabolic capacity, or in individuals with gene variants that are associated with the reduced expression or compromised function of key enzymes, habitual drug use may be important.

### Impact of habitual diet composition on flavonoid ADME

The impact of habitual dietary intake on flavonoid bioavailability has not been extensively investigated. However, the effects of alcohol, fiber, and dietary fat composition have been studied to a limited degree. In a recent study, differences in microbial metabolite concentrations in feces were measured after intake of either red wine or dealcoholized red wine (92) with the suggestion that the alcohol content may increase the solubility of the polar flavonoid compounds. However, no significant differences in total metabolite amounts were observed. In other studies, although no difference in plasma catechin concentrations were observed after intake of either red wine or dealcoholized red wine, the urinary excretion of catechins was more rapid after red wine intake (93, 94).

The impact of fiber intake on flavonoid bioavailability is not clearly understood and, to our knowledge, has not been investigated in human studies. It has been speculated that a high fiber content may decrease the availability and bioaccessibility of flavonoids from the foods because of factors such as physical entrapment, increased viscosity, and increased bulk (95). However, because of the impact of the microbiome on flavonoid metabolism, the potential bidirectional relation (see Impact of the gut microbiome on flavonoid metabolism) together with the established effects of fiber intake on intestinal transit time and short-chain fatty acid (SCFA) production, there is the potential to enhance flavonoid bioavailability and metabolism in the large intestine. In mice, pectin enhanced quercetin absorption, which was likely the result of an alteration in the metabolic activity of the microbiome (96). Although, to our knowledge, no systematic studies exist for flavonoids, a reduced gastrointestinal transit time has been shown to decrease the bioavailability of various drugs (97). To date, few studies have investigated the effect of dietary fat intakes on flavonoid absorption. Because most polyphenols are water soluble and transported via the portal vein, dietary lipids are likely to have little influence on the more-hydrophilic flavonoids. However, there may be important interactions with the more hydrophobic (lower number of hydroxyl groups) flavonoids (98). Dietary fats also alter the gastrointestinal transit time, and this variation has the potential to alter flavonoid kinetics and absorption. In an acute human study, strawberries, when consumed with cream, delayed the excretion of anthocyanin metabolites in the first 2 h but did not alter the total bioavailability (as measured by the AUC in plasma) of anthocyanins (99). In an in vitro digestion model, the higher fractional bioaccessibility of procyanidins, but not of phenolic acids and flavones, was observed from lipid-rich cacao liquor (45% fat) than from cacao powder (15% fat) (100), thereby supporting the notion that polar flavonoids are, at least in part, micellularized and that lipids from the food matrix may help in stabilizing the mixed micelles or in making them more soluble. Although a clear dose-response effect was not evident, an improved bioavailability of quercetin was observed in pigs fed quercetin together with test meals that differed in fat contents (3, 17, or 32 g fat/100-g diet). The AUC after the 17% fat diet was ∼57% higher than that of the 3% fat diet, with no further increase shown when a 32% fat diet was fed (101). In a small human study (*n* = 9), the AUC in plasma quercetin concentrations was 45% higher in subjects who consumed a fat-rich breakfast than in subjects who consumed a fat-free breakfast (102). In mice, Giunta et al. (103) showed that fish oil and green tea–derived (−)-epigallocatechin-3-gallate (flavan-3-ols) had a significantly greater antiamyloidogenic effect than that of either component fed separately. The inclusion of fish oils (rich in n–3 fatty acids) in the rodent diet increased both blood and brain (−)-epigallocatechin-3-gallate concentrations.

As well as the potential impact of a habitual diet on metabolism, note that other sources of variability include the wide variation in the flavonoid contents of foods. In epidemiologic studies, food-composition databases have been used to assess intake, which have not accounted for the variability in content that occurs as a result of different growing conditions and processing and cooking techniques. However, despite these sources of variation, observational data have allowed us to rank order intakes, thereby allowing comparisons between high and low intakes in large population groups. Until validated biomarkers that integrate intake with in vivo metabolism are available, current data can only be derived from dietary intake information. In many trials, the wide variability in the flavonoid contents of intervention foods is often not considered, and an independent verification of the amounts that are present in intervention foods and supplements would allow for a more accurate assessment of the dose-response effects in the future.

### Impact of the physiochemical properties of food on flavonoid metabolism

In relation to the impact of food composition, Brett et al. (104) observed no differences in the absorption and excretion of flavanones after feeding a whole-fruit matrix compared with an orange-juice matrix. However, the solubility of flavanones are thought to be a key factor for bioavailability, and in juices that contained different flavanone concentrations, higher urinary excretion and plasma concentrations were associated with soluble flavanone concentrations in the juice (105). Although there was little intraindividual variability, the interindividual variability was large, which supported the notion that specific microbiota are required to cleave the glycosides (rutinosides) in the juice, thereby resulting in aglycones that are available for absorption (105). However, more-recent data have suggested that, although food-processing methods to improve solubility can enhance bioavailability, the stratification of volunteers relative to their excretion capabilities was more important (105, 106).

The flavonoid sugar moiety has been suggested to be an important determinant for both the absorption site and overall bioavailability in humans (107, 108). One of the most predominant forms is the attachment to a β-glucoside, which can only be absorbed to a very limited extent, and needs to be hydrolyzed before absorption in the small intestine (see Absorption) (7). For flavonoids with other additional attachments, including rhamnose (the flavonol quercetin), the microflora are required to cleave off the sugar moieties before absorption (109). After enzymatic treatment with rhamnosidase, the rutinoside moiety can be hydrolyzed to produce the glucoside moiety, and after enzyme treatment, the bioavailability of flavanones from juice has increased 4-fold in humans (110). Moreover, it is not only the chemical structure but also their isomeric configuration that can affect absorption. For the metabolism of (R/S) hesperidin, hesperitin-7-glucoside was shown found to have an R:S ratio of 39:69 in human plasma and urine samples, thereby suggesting that the S configuration could be more bioavailable (111). Specifically for flavan-3-ols, the bioavailability may be influenced by the differing proportions of the various enantiomeric forms of the monomeric flavan-3-ols. Unlike other flavonoids, flavan-3-ols exist in plants as aglycones rather than as the glycoside form. In one study in which comparable concentrations of the individual enantiomers were separately consumed, the bioavailability of the different stereoisomers differed widely (112), but in most trials, the individual profile of flavan-3-ol stereoisomers has been rarely characterized but may be one factor that may explain differences in the bioavailability and bioactivity across published human studies.

### Impact of the gut microbiome on flavonoid metabolism

Colonic metabolism has long been speculated to be a major contributor to the overall metabolism of not only dietary flavonoids but also of phase I and II metabolites that have been excreted back into the intestine via enterohepatic circulation (10, 113). The microbial metabolism of flavonoids is thought to follow a general pattern whereby a diverse range of compounds are funneled to a reduced number of metabolites. The bacterial enzymes deglycosylate the compounds, but the microbes can also perform a range of other transformations including oxidation, demethylation, and the catabolism to smaller fragments including small phenolic acids and aromatic catabolites (7, 8, 114–116). However, it remains unclear how well these metabolites are absorbed. The colonic bioconversion of flavonoids is thought to be highly variable although the etiology of the heterogeneity is currently unclear. There is wide interindividual variability in the bioconversion of specific flavonoids (115, 117, 118) that has been attributed in part to specific enterotypes and has resulted in the suggestion that individuals may be either low- or high-flavonoid convertors (106, 119). The interindividual variability may also be related to the fact that small differences in the chemical compositions of flavonoids (substitution patterns) can result in major changes in colonic bioconversion (119) or the modulation of the flavonoid-microbiota interaction by the background habitual diet, which varies dramatically across population groups (120). Many of these microbiome-mediated chemical transformations can result in the production of metabolites with increased biological activity, with the most-notable example being the isoflavones, which are a subclass of flavonoids that are derived predominately from soy. In the 1980s, evidence that the microbiome was key for metabolism to the specific microbial-derived metabolite equol emerged (121), and wide interindividual variability in the ability to produce this microbial-derived metabolite has been established, ranging from 25–30% equol producers in Western populations to 50–70% in Asian counties (122–124). Equol has been shown to be more bioactive than its food precursor daidzein in vitro and in trials (predominantly with the equol-producer phenotype assessed retrospectively), the magnitude of the biological effect was greatly enhanced in participants who produced equol after isoflavone ingestion, which suggested that there is a critical role of the microbiome for health effects (125–127). In general terms, there is emerging literature that describes the diverse and significant impact of flavonoid phenolics and other small molecules that are produced in the large intestine on physiologic processes such as SCFA production and bioavailability, bile acid metabolism, redox and inflammatory status, and associated intestinal, hepatic, and overall systemic functions (128). The production of SCFA is of interest in colonic health, dietary energy extraction, and body-weight regulation (128, 129). Although flavonoid-induced effects on the main SCFAs acetic (C2), propionic (C3), and butyric (C4) acids have been repeatedly shown in in vitro fermentation systems and in rodent models (128), data from human interventions have been limited and nonconclusive. For example, in healthy humans, red-wine grape-juice extract, but not grape-juice extract, fed for 4 wk reduced fecal isobutyric acid concentrations (130), whereas Mosele et al. (131) reported no impact of 4 wk of pomegranate-juice consumption on total or individual fecal SCFA concentrations.

The large observed heterogeneity in the bioactivity and bioavailability of different flavonoid metabolites that are formed after ingestion, including the extensive range of the microbial-derived metabolites identified particularly for anthocyanins (11, 116), supports a strong interplay between flavonoids and the microbiome. Although it is likely that flavonoid intake alters the composition and function of the gut microbiome, and, conversely, microflora enhances the metabolism of flavonoids, this bidirectional relation has not yet been addressed in flavonoid research to our knowledge. An examination of this bidirectional relation has been limited to a few small cross-sectional or short-term feeding studies. A cross-sectional study, which included 178 elderly subjects, observed that habitual diet-driven microbiota alterations were associated with health status, including measures of frailty and inflammation (132), whereas in a study that was limited to 15 women, a 2-mo dietary intervention was associated with changes in *Gammaproteobacteria* and *Erysipelotrichi* microbial communities (133). In a recent small RCT (*n* = 9), high amounts of *Bifidobacteria* were associated with increased amounts of flavonoid microbial metabolites after polyphenol-rich wine intake (134). Several recent animal studies observed profound effects in the gut microbial community structure after intake of flavonoid-rich foods although it is possible that there are differences in the permeability of microbiome-derived metabolites between rodents and humans. In one animal study, a reduction in the ratio of *Firmicutes* to *Bacteroidetes* and an increase in *Akkermansia muciniphila* were observed after intake of grape extract; these changes conferred protection against the negative consequences of a high-fat diet, which resulted in a reduction in inflammation and an improvement in insulin sensitivity (135). An additional study in mice showed that the microbial composition (specifically *Akkermansia* spp.) played a decisive role in the observed protective effects of a cranberry extract from diet-induced obesity and insulin resistance (136). Other flavonoid-rich foods, including green and black teas, have also been shown to increase the proportion of *Akkermansia* (137, 138). These animal data provide the first convincing data that the gut microbiome may play a substantial role in mediating the health effects of flavonoids, thereby leading to a reduction in inflammation and improved metabolic function (135, 136). It remains to be determined in humans if the interaction between flavonoids and the gut microbiota is a direct effect or an indirect effect (mediated through altered host physiology), but these animal data provide clear evidence of significant interactions. The similarity in animal responses to different sources of flavonoids (grape and cranberry) also suggest that perhaps diverse sources of flavonoids may have similar effects on the gut microbiome, but it is only through human intervention trials that the significance to human physiology can be established.

In humans, we know that in the subclass anthocyanins, after feeding stable-isotope labeled anthocyanins, they are extensively degraded, which is swiftly followed by further transformation (11, 116). These data provide some support from human data that anthocyanin bioactivity is likely mediated by the high concentrations and longer half-lives of its microbial-derived phenolic metabolites (116). Recent research has suggested that, in vitro, nutritionally relevant amounts of these colonic metabolites exert greater vascular and anti-inflammatory activity than do the metabolites that are formed and absorbed in the small intestine (139–141), thereby providing additional evidence that the bioactivity of anthocyanins is highly likely attributed to their microbial-derived metabolites. Clinical studies to determine whether these effects are also observed in humans are urgently needed because the identities of the main microbiota phyla and species that modulate anthocyanin metabolism in humans are unknown, and data from adequately powered acute studies and long-term human RCTs investigating the potential of microbiota diversity to explain associations between anthocyanin intake and CVD risk are completely lacking.

### Potential impact of gut-immune homeostasis and intestinal permeability on flavonoid ADME

The intestinal epithelium, together with the colonic bacteria, is the first site of interactions between food intake and the host immune system, and this interaction can affect the microbiota composition, which, in turn, can directly affect gut-immune homeostasis and intestinal permeability and potentially flavonoid ADME. If flavonoids are acting primarily at the level of intestinal absorption, an understanding of how different dietary flavonoids influence and regulate the intestinal barrier and intestinal permeability is key. In 2 pivotal animal studies, profound effects of flavonoid intake on the microbial community structure were observed with resulting effects on intestinal and systemic inflammation and the metabolic response (135, 136). However, overall, the data suggested that these effects were the result of a direct trophic influence of the flavonoids on *Akkermansia* rather than an effect on mucin production. In one study, mucus production was increased, but the authors suggest that this increased production may have followed the direct effects of flavonoids on *Akkermansia* (136), whereas in another study, no differences in mucin gene expression in jejunum or colon samples were observed (135). A direct effect on increasing the abundance of *Akkermansia* fits with other in vitro data (138).

The relative increase in *Akkermansia* after cranberry intake was also associated with the prevention of a high fat– and high sugar–induced rise in liposaccharide and a decrease in intestinal inflammation (136). These observations suggest that, by increasing *Akkermansia,* flavonoids may reduce intestinal permeability and liposaccharide leakage, thereby ameliorating insulin resistance in diet-induced obese mice (136). The understanding of such interactions in humans is a key next step.

Because large proportions of ingested flavonoids reach the colon and undergo extensive bioconversion, it is likely that the resultant metabolites exert local intestinal effects while in the colon and systemic effects after absorption. In vitro, the flavonol quercetin was shown to enhance barrier function in rat small and large intestines and exerted protective effects on cytokine-induced barrier damage. In caco-2 cell monolayers, several flavonoids, including flavanols and flavanones, exerted beneficial effects on intestinal barrier function (increased epithelial resistance and claudin-4 expression in epithelial cells) (142–144). The impact of these localized effects of flavonoids in the colon on flavonoid bioavailability remains to be established.

### Concluding remarks

In summary, although there is growing evidence from prospective cohort studies and clinical trials of the potential health benefits of dietary flavonoids, this review highlights the research gaps in the current knowledge base (**Text Boxes 1** and **2**).

Text Box 1 **Research challenges and future studies required in flavonoid research**

- Conduct adequately powered clinical studies to determine the impact of age, sex, habitual diet, genotype, drug interactions, and the microbiome on flavonoid metabolism.
- Conduct trials to understand the bidirectional relation between flavonoid metabolism and the microbiome.
- Prospectively recruit participants to clinical trials on the basis of the extent of absorption and metabolism to establish dose-response relations.
- Identify and validate a panel of robust biomarkers of flavonoid intake and subsequent metabolism that can be used to examine associations of bioavailable flavonoids with health outcomes in future prospective cohort studies.
- Further develop metabolomic data sets to assist in the development of biomarkers.
- Conduct hypothesis-driven research to investigate the impact of specific genotypes on flavonoid metabolism with a particular focus on variants in LPH, β-glucosidases, phase I metabolism, and phase II metabolism with prospective recruitment by genotype for associations established with the use of the retrospective genotype approaches.
- Conduct intervention studies to determine how food composition and flavonoid source affect bioavailability.
- Conduct trials in which metabolism and health outcomes are addressed simultaneously.

Text Box 2 **Study design and research considerations**

- Conduct longer-term intervention trials (≥6 mo) and consider long-term trials with clinical outcomes.
- Conduct head-to-head comparisons of flavonoid extracts and pure compounds compared with flavonoid-rich foods in clinical trials.
- In animal-model experiments, give consideration to the dose fed to ensure it is applicable to human intake.
- In examining mechanistic insights in vitro, consider the use of physiologically relevant doses and focus on metabolites.
- Develop high-throughput assays for assessing metabolites and develop appropriate standards for mass spectrometry to quantify the range of metabolites produced in vivo.
- Develop optimal placebo products for clinical trials.
- Select the genotyping approach with close consultation with a genetics expert for a limited targeted genotyping of specific genes of interest, nonsynonymous variants, or variants in the promoter region of the gene that are most likely to be functional.
- Once genotype-metabolism associations have been established with the use of retrospective genotyping approaches, confirm the impact of the genotype *1*) in an independent human study with the use of prospective recruitment on the basis of genotype and *2*) in rodent transgenic models expressing the human variant versions of the gene.

At a population level, the heterogeneity in responsiveness to habitual flavonoid intake obscures beneficial associations between intakes and health outcomes in responsive population subgroups and creates a difficulty in establishing the physiologic and molecular mechanisms that underlie the health benefits of different flavonoid subclasses. Identifying key factors governing metabolism and understanding if a differential capacity to metabolize these bioactive compounds affects health outcomes will greatly enhance the ability to optimize intakes of flavonoids for health benefits. The large observed heterogeneity in the bioactivity and bioavailability of different flavonoid metabolites that are formed after ingestion, including the extensive range of microbial-derived metabolites identified, supports a strong interplay between flavonoids and the microbiome. Although it is likely that flavonoid intake alters the composition and function of the gut microbiome, and conversely, microflora enhances the metabolism of flavonoids, this bidirectional relation has not been addressed in clinical trials to our knowledge. Furthermore, we have identified a dearth of data on genetic determinants of flavonoid metabolism. Although it is thought that ≥50% of the interindividual variability in ADME may be attributed to genetic variability, little research focus has investigated which gene variants may alter flavonoid ADME. An understanding of the impact of compromised phase I and phase II metabolism that are mediated by genotype or variables such as age, sex, or habitual prescription drug intake on flavonoid bioavailability and metabolism is almost completely lacking. Addressing these research gaps (Text Boxes 1 and 2) would provide the basis for the development of targeted dietary advice for subgroups who are likely to be most responsive and help us work toward the development of specific dietary guidelines for several dietary flavonoid subclasses. These research gaps build on the guidance and key considerations for the design and reporting in flavonoid research that were outlined by Balentine et al. (145) in 2015. In prospective studies, an understanding of interindividual variation after flavonoid intake would allow the establishment of validated biomarkers that are indicative of both flavonoid intake and subsequent metabolism to further establish relations between bioavailable flavonoids and health outcomes.

## Footnotes

2 Abbreviations used: ABC, ATP-binding cassette; ADME, absorption, distribution, metabolism, and elimination; COMT, catechol-*O*-methyltransferase; LPH, lactase phlorizin hydrolase; RCT, randomized controlled trial; SCFA, short-chain fatty acid; SNP, single-nucleotide polymorphism; UGT, urine-5′-diphosphate glucuronosyltransferase.
